# The hidden regulators: Non‐coding RNAs in *KMT2A* ‐rearranged acute lymphoblastic leukemia

**DOI:** 10.1002/ijc.70283

**Published:** 2025-12-08

**Authors:** Maria Augusta Poersch, Ana Carolina Rodrigues, Priscila Elias Ferreira Stricker, Alexandre Luiz Korte Azevedo, Daniel Pacheco Bruschi, Jaqueline Carvalho de Oliveira

**Affiliations:** ^1^ Genetics Post‐Graduation Program, Genetics Department, Federal University of Parana Curitiba Parana Brazil

**Keywords:** acute lymphoblastic leukemia, KMT2A rearrangement, mixed lineage leukemia, non‐coding RNAs

## Abstract

Acute lymphoblastic leukemia (ALL) driven by *KMT2A* rearrangements (*KMT2A*‐r) is an aggressive hematologic malignancy with poor prognosis and a high incidence in infants. While KMT2A fusion proteins drive leukemogenesis through transcriptional dysregulation, recent discoveries have highlighted the pivotal role of non‐coding RNAs (ncRNAs) in shaping the molecular and epigenetic landscape of this disease. These key regulators of gene expression influence chromatin dynamics, transcriptional activation, and post‐transcriptional control. Circular RNAs (circRNAs) contribute to genome instability and facilitate chromosomal translocations, while some fusion‐derived circRNAs (f‐circRNAs) sustain oncogenic signaling and promote chemoresistance. Long non‐coding RNAs (lncRNAs) orchestrate transcriptional programs that maintain leukemic stem cell properties and reinforce aberrant self‐renewal pathways. MicroRNAs (miRNAs) modulate critical oncogenic networks by regulating KMT2A fusion transcripts and downstream effectors, thereby impacting drug resistance, apoptosis, and proliferation. Meanwhile, enhancer RNAs (eRNAs) fine‐tune transcriptional activity and epigenetic regulation, influencing KMT2A target gene expression and chromatin accessibility. Collectively, these ncRNAs integrate into the complex regulatory circuits of *KMT2A*‐r ALL, revealing their potential as biomarkers for disease classification, risk stratification, and treatment response prediction. Understanding their interplay with KMT2A fusion proteins not only provides new insights into leukemogenesis but also highlights promising opportunities for therapeutic intervention and precision medicine in this high‐risk leukemia subtype.

## Graphical abstract

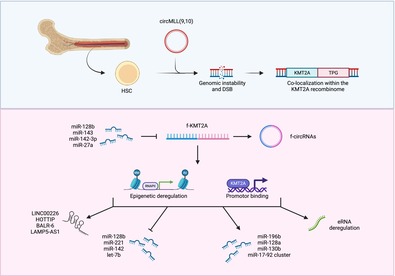

**Abbreviations**
ALL: Acute lymphoblastic leukemia
BALR: B‐ALL Associated Long RNAs
BCR: break cluster region
ChIP: Chromatin immunoprecipitation
circRNA: circular RNA
DSB: double‐strand break
ER3D: Enhancer RNA‐mediated Recombination of 3D Genomic Elements
eRNA: enhancer RNA
f‐circRNAs: fusion circRNAs
H3K4me3: histone H3 lysine 4 trimethylation
HSC: hematopoietic stem cell
KMT2A‐CPs: KMT2A chimeric proteins
*KMT2A*‐r: *KMT2A* rearranged
lncRNA: long non‐coding RNA
miRNA: microRNA
MLL: Mixed lineage leukemia
mRNA: messenger RNA
ncRNA: non‐coding RNA
RIP: RNA immunoprecipitation
rRNA: ribosomal RNA
snoRNA: small nucleolar RNA
snRNA: small nuclear RNA
ssDNA: single‐stranded DNA
TPG: translocation partner gene
tRNA: transfer RNA

## 1 INTRODUCTION

Leukemia is the most common neoplasm in children, accounting for approximately one‐third of pediatric cancer cases worldwide.^1^, ^2^ Among its subtypes, acute lymphoblastic leukemia (ALL) is the most frequent, representing about 80% of diagnoses.^1^, ^3^ ALL is a complex and heterogeneous neoplasm that continuously challenges the scientific community due to its molecular variations and clinical outcomes that rely on molecular subgroups defined by translocations, hyperdiploidy, and other genetic alterations.^4^, ^5^, ^6^ These subgroups are critical for risk stratification in ALL, categorizing cases as low, intermediate, or high risk.^5^, ^6^

Leukemia driven by rearrangement of the lysine methyltransferase 2A (*KMT2A*) gene, previously known as mixed lineage leukemia (*MLL*), is characterized by a rapid onset and aggressive progression, being one of the most important predictors of adverse outcomes in childhood leukemia.^7^, ^8^, ^9^ Although these rearrangements are less common in older children and adults, they are the leading cause of infant ALL, with 70‐80% of cases harboring this alteration.^6^, ^10^, ^11^, ^12^

*KMT2A‐r* disrupts normal cell behavior through epigenetic changes and gene deregulation, activating stem cell pathways and gene clusters such as *HOXA* and *MEIS1*.^13^, ^14^, ^15^, ^16^ Beyond the important role of *KMT2A*‐r in deregulating significant genetic networks with known coding genes, the role of non‐coding RNAs (ncRNAs) cannot be overlooked in this context.^16^, ^17^, ^18^, ^19^ The majority of the genome is transcriptionally active, producing a diverse array of ncRNAs with essential functions.^20^, ^21^, ^22^ Notably, the prevalence of ncRNAs correlates with organismal complexity, highlighting their crucial role in the intricate regulatory networks required for multicellular life.^20^, ^23^, ^24^ The role of certain ncRNAs, such as long non‐coding RNAs (lncRNAs), in the dysregulation of B‐cell ALL is well‐established.^25^, ^26^, ^27^, ^28^ These molecules offer valuable insights into prognosis and treatment response, making them promising biomarkers for risk stratification and disease classification.^29^, ^30^, ^31^

Given the critical role of ncRNAs in gene regulation and their emerging significance in leukemia, this review examines the molecular mechanisms underlying *KMT2A* rearrangements and key ncRNAs identified and characterized in its regulation, focusing on the regulatory networks they participate in, their mechanisms of action, and their potential as biomarkers and therapeutic targets in *KMT2A*‐r ALL.

## 2 THE *KMT2A* GENE AND ITS TRANSLOCATION PARTNER GENES

The *KMT2A* gene encodes a large, multi‐domain protein that serves as both a transcriptional activator and an epigenetic regulator.^32^, ^33^ Under regular physiological conditions, KMT2A plays a crucial role in hematopoiesis, embryonic development, stem cell differentiation, adipogenesis, circadian gene regulation, and responses to external stimuli.^33^, ^34^, ^35^, ^36^, ^37^, ^38^ Depending on the methylation site, KMT2A can either activate or repress transcription, and it is essential for the development and maintenance of hematopoietic stem cells (HSCs).^32^, ^33^, ^34^, ^39^, ^40^

**FIGURE 1:**
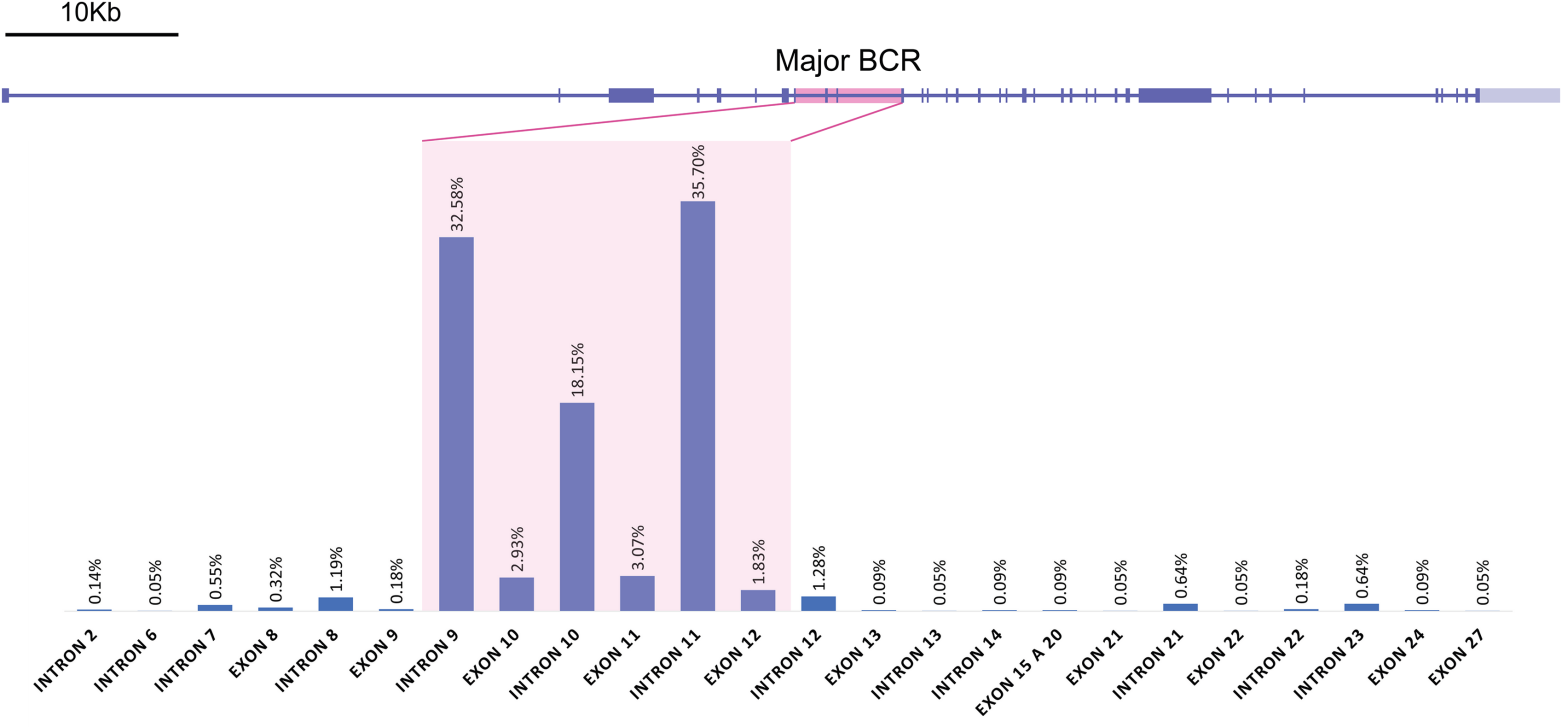
Schematic representation of the *KMT2A* gene, with the major BCR highlighted in pink. Below, the percentage of annotated breakpoints within different exons and introns of *KMT2A*, adapted from Meyer et al.^8^

However, in the context of ALL, chromosomal translocations involving *KMT2A* and various translocation partner genes (TPGs) can result in the formation of chimeric proteins that profoundly impact tumorigenesis.^41^ Over 90% of *KMT2A* breakpoints occur between exons 9 and 12 (Figure 1), often resulting in reciprocal fusion proteins with a variety of TPGs.^8^ Among these, the most frequently reported partners are *AFF1*, *MLLT1*, *MLLT3*, *MLLT10*, *AFDN*, and *EPS15*, which together account for more than 90% of cases. *AFF1* is the most prevalent TPG, occurring in approximately 46% of cases.^8^

Most of *KMT2A*'s TPGs are primarily associated with transcription initiation and elongation complexes, being important regulators of RNA polymerase II activity.^42^ Due to the nature of these translocations and the function of the genes involved, KMT2A chimeric proteins (KMT2A‐CPs) typically retain promoter‐binding and reading domains, along with the potent transcription activation domains derived from the fusion TPG.^43^ As a result, these CPs recruit elongation protein complexes and activate target gene transcription.^43^, ^44^

As these chimeric proteins interact with various genetic networks, they can lead to the activation of key target genes; among these, *HOXA* transcription factors (particularly *HOXA9*), *MEIS1*, *PBX3*, *CDK6*, and *MEF2C* stand out, playing significant roles in the disease's progression.^9^ In this context, ncRNAs also play a critical role in the regulation of *KMT2A*‐r molecular networks, often activating genomic sequences associated with enhancers and oncogenes, further contributing to leukemogenesis.^45^, ^46^

In the following sections, we will provide an overview of ncRNAs and delve into the most important ncRNAs implicated in *KMT2A*‐r leukemia.

## 3 NON‐CODING RNAs

Broadly defined as RNA molecules that do not encode proteins, ncRNAs can be categorized into two major groups based on their function: housekeeping and regulatory.^47^, ^48^ Housekeeping ncRNAs, such as transfer RNAs (tRNAs) and ribosomal RNAs (rRNAs), are essential for protein synthesis, while small nucleolar RNAs (snoRNAs) guide chemical modifications of other RNAs, and small nuclear RNAs (snRNAs) regulate splicing events.^49^, ^50^

In contrast, regulatory ncRNAs control gene expression at all known levels.^50^, ^51^ These ncRNAs can be further classified based on their length, structure, and cellular localization, and include small regulatory RNAs such as microRNAs (miRNAs) and PIWI‐interacting RNAs (piRNAs), as well as long non‐coding RNAs (lncRNAs) that modulate gene expression at transcriptional, post‐transcriptional, and epigenetic levels.^52^, ^53^, ^54^, ^55^ This expanded understanding of the functional RNA landscape identified ncRNAs as critical players in cellular complexity and innovation, underscoring the need for further investigation into their origins, roles, and therapeutic potential.^48^, ^53^

NcRNA‐mediated gene regulation is highly cell type‐ and context‐dependent, with the same molecule capable of acting as a tumor suppressor in one cancer type while promoting disease progression in another.^56^ Despite being as essential to cellular function and development as proteins,^57^ ncRNAs remain poorly understood in the context of *KMT2A*‐r leukemia. Although existing research suggests that ncRNAs hold promise as biomarkers, prognostic indicators, and therapeutic targets (Table 1), much of this knowledge remains insufficiently disseminated.^58^, ^59^, ^60^, ^61^

**TABLE 1:** Overview of dysregulated and fusion‐associated ncRNAs in *KMT2A*‐r leukemia.

Class	Name	Status	References
circRNA	circMLL(9,10)	Upregulated	59
circRNA	f‐circM9_1	Fusion‐derived	66
circRNA	circAFF1	Fusion‐derived	67
lncRNA	LAMP5‐AS1	Upregulated	69, 70
lncRNA	LINC00226	Upregulated	69
lncRNA	LINC01221	Upregulated	69
lncRNA	ENST00000418618	Upregulated	69
lncRNA	LINC01226	Upregulated	69
lncRNA	CASC15	Upregulated	71
lncRNA	BALR‐2	Upregulated	28
lncRNA	BALR‐6	Upregulated	28, 72
lncRNA	HOTTIP	Upregulated	73
miRNA	miR‐128b	Downregulated	83, 84
miRNA	miR‐221	Downregulated	83
miRNA	miR‐128a	Upregulated	79
miRNA	miR‐130b	Upregulated	79
miRNA	miR‐143	Downregulated	85
miRNA	miR‐142 (miR‐142‐3p)	Downregulated	77, 85
miRNA	miR‐205	Regulatory	85
miRNA	miR‐27a	Downregulated	86
miRNA	miR‐196b	Upregulated	87, 88, 89
miRNA	let‐7b	Downregulated	78, 91
miRNA	miR‐17‐5p	Upregulated	61, 90
miRNA	miR‐17‐3p	Upregulated	61
miRNA	miR‐18a	Upregulated	61
miRNA	miR‐19a	Upregulated	61, 90
miRNA	miR‐19b	Upregulated	61
miRNA	miR‐20a	Upregulated	61
miRNA	miR‐92	Upregulated	61
eRNA	SEELA1	Upregulated	98
eRNA	SEELA2	Upregulated	98
eRNA	Myrlin	Enhanced activity	60

### 3.1 circRNAs

Circular RNAs (circRNAs) are a unique class of non‐coding RNA molecules distinguished by their covalently closed loop structure, formed through back‐splicing events.^62^ Unlike linear RNAs, circRNAs are highly stable, resistant to exonuclease degradation, and often enriched in specific cell types or developmental stages, suggesting a functional role in gene regulation and disease pathology.^51^, ^62^ One intriguing mechanism by which circRNAs contribute to genome instability is through the formation of circR loops, where circRNAs hybridize with complementary genomic DNA sequences.^59^, ^63^, ^64^ This interaction exposes single‐stranded DNA (ssDNA) regions vulnerable to double‐strand breaks (DSBs) and mutations.^59^, ^63^ Such events are particularly critical in the *KMT2A* recombinome, a genomic region enriched with circRNA‐producing genes, where circRNAs can act as facilitators of chromosomal translocations.^59^ The novel mechanism, termed enhancer RNA‐mediated Recombination of 3D Genomic Elements (ER3D), suggests that circRNAs, by forming stable circR loops, amplify the risks of genome instability and oncogenic rearrangements.^59^

In the specific context of *KMT2A‐r* leukemia, recent evidence highlights a direct link between circRNAs and the disease's pathogenesis. Conn et al.^59^ reported that neonatal blood samples from eight healthy patients who later developed *KMT2A‐r* leukemia exhibited markedly elevated levels of the circular RNA circMLL(9,10)—up to 100 times higher compared to healthy controls—even before the chromosomal translocation event occurred. Mechanistically, circMLL was shown to pause RNA polymerase II (RNAPII) at the break cluster region (bcr) of the *KMT2A* gene. This stalling promotes the accumulation of transcriptional stress, leading to DSBs and facilitating chromosomal translocations. Furthermore, circRNAs promote the co‐localization of loci within the *KMT2A* recombinome, suggesting that circMLL actively drives the genomic rearrangements characteristic of *KMT2A*‐r leukemia, rather than being a mere byproduct of oncogenic processes.^59^ Understanding how circRNAs interact with the *KMT2A* locus and how the bcr is enriched in circRNAs pinpoints the potential of circMLL as an early biomarker for leukemia predisposition.

Beyond its role in initiating chromosomal translocations, the impact of circRNAs in *KMT2A*‐r leukemia extends to their influence on the three‐dimensional genome architecture. By interacting with enhancer elements and regulatory proteins, circRNAs may rewire transcriptional networks, further amplifying oncogenic signals.^59^, ^62^ This complex interplay between circRNAs, genomic instability, and transcriptional regulation positions circRNAs as central players in leukemogenesis and highlights their potential as both biomarkers and therapeutic targets.

Most of the 90 *KMT2A* translocation partners express circRNAs, with 54 genes of the recombinome expressing at least one abundant circRNA.^65^ Notably, in cases of *KMT2A* translocations, fusion circRNAs (f‐circRNAs) can be produced as a consequence of chromosomal translocations. The breakpoint position defines which genomic regions are retained in the fusion gene, determining not only if and how f‐circRNAs are generated but also affecting the expression levels of circRNAs normally generated by *KMT2A* and its partner genes. Unlike their linear mRNA counterparts, f‐circRNAs are not degraded by conventional nonsense‐mediated decay pathways, giving them enhanced stability and persistence within cells. Studies indicate that they have the potential to act as oncogenic drivers, contributing to leukemogenesis by promoting cellular transformation, enhancing proliferation, and fostering therapeutic resistance.^65^

One notable example is f‐circM9_1, derived from the *KMT2A*::*MLLT3* fusion gene.^66^ Functional studies have demonstrated its ability to induce oncogenic characteristics in vitro and in vivo. In mouse fibroblast models, f‐circM9_1 expression was shown to promote tumor proliferation, underscoring its role in the initiation and progression of leukemia. Furthermore, cells expressing f‐circM9_1 exhibited resistance to chemotherapeutic agents such as arsenic trioxide and cytarabine. This resistance is thought to stem from the f‐circRNA's influence on apoptosis‐related pathways, as these cells displayed enhanced survival rates and lower apoptotic activity when exposed to these treatments. The capacity of f‐circM9_1 to modulate therapeutic response makes it a critical target for understanding disease progression and identifying new treatment strategies.^66^

Aberrant expression patterns of f‐circRNAs associated with the *KMT2A*::*AFF1* fusion gene (circAFF1) have also been implicated in the aggressive nature of *KMT2A*‐r leukemia.^67^ Unlike linear fusion transcripts, circAFF1 operates through distinct mechanisms that enhance leukemic cell proliferation and survival. Functional experiments revealed that circAFF1 interacts with important signaling pathways, possibly by sequestering miRNAs or RNA‐binding proteins, which may enhance the stability and translation of pro‐oncogenic mRNAs. This underscores the active role of fusion circRNAs in shaping the leukemic phenotype and highlights their contribution as more than byproducts of chromosomal translocations.^67^

These findings highlight the importance of circRNAs as one more layer in the molecular mechanisms of *KMT2A*‐r leukemia. The ability of circRNAs to promote chromosomal translocations and alter transcriptional dynamics emphasizes their dual role as both drivers and regulators of oncogenesis.

### 3.2 lncRNAs

The majority of animal genomes are dynamically transcribed into lncRNAs, which have little or no protein‐coding potential.^47^, ^52^ These transcripts are typically generated by RNA polymerase II and are classified as being at least 500 nucleotides in length.^52^ Characterized lncRNAs have been shown to play roles at virtually every level of genome organization, cell structure, and gene expression regulation across multiple levels, including epigenetic, transcriptional, and post‐transcriptional processes.^52^, ^68^ Over 100,000 human lncRNAs have been identified, many of which are primate‐ and cell type‐specific.^52^ Yet this is likely an underestimation, as many remain uncharted across developmental stages and rare cell types.^52^

In *KMT2A*‐r ALL, the regulatory landscape of lncRNAs is only beginning to unfold; nevertheless, it already reveals patterns of subtype specificity and mechanistic complexity.^28^, ^69^ One of the earliest studies comparing *KMT2A*‐wildtype and *KMT2A*‐rearranged samples via microarray uncovered distinct lncRNA signatures unique to each rearranged fusion subtype, including *KMT2A*::*AFF1*, *KMT2A*::*MLLT3*, and *KMT2A*::*MLLT1*, as well as differences between infant and older patients.^69^ Among the most upregulated transcripts were LAMP5‐AS1, LINC00226, and LINC01221, with expression levels later validated by RT‐qPCR.^69^

Of particular interest, LAMP5‐AS1 was shown to enhance the activity of DOT1L, a histone methyltransferase responsible for H3K79 dimethylation and trimethylation, indirectly activating gene programs that sustain leukemic stemness.^70^ These findings place LAMP5‐AS1 at the intersection of transcriptional control and epigenetic regulation, positioning it as a candidate for targeted therapy. Inhibiting LAMP5‐AS1 or DOT1L disrupts self‐renewal pathways essential to *KMT2A*‐r leukemia progression.^70^

Building on this regulatory axis, a subset of lncRNAs was found to correlate positively with *HOXA* gene expression. Silencing two of the identified lncRNAs, *ENST00000418618* and *LINC01226*, in the MV4‐11 cell line led to decreased proliferation and increased apoptosis,^69^ supporting a functional role in leukemic maintenance. Additional mechanistic insight into lncRNA function came from the study of *CASC15*, whose knockdown in RS4;11 cells reduced prednisolone‐induced apoptosis—possibly due to downregulation of *SOX4*, a transcription factor critical for B‐cell development.^71^ This suggests that *CASC15* may influence treatment response, raising its profile as a potential biomarker for therapy stratification.

Broader transcriptomic analyses further highlighted the diagnostic potential of lncRNAs.^28^ A comparative study across B‐ALL cytogenetic subtypes identified four highly expressed lncRNAs, B‐ALL Associated Long RNAs (BALRs) *BALR‐1*, *BALR‐2*, *BALR‐6*, and *LINC00958*. Among them, *BALR‐2* was linked to poor prognosis and reduced responsiveness to prednisone. Its knockdown in RS4;11 cells triggered apoptosis and reduced proliferation while inducing JUN and BIM, echoing the transcriptional effects of glucocorticoid treatment.^28^ *BALR‐6*, on the other hand, functions as a pro‐survival factor, with loss‐of‐function studies showing that its depletion impairs proliferation and increases apoptosis, while in vivo overexpression disrupts hematopoietic differentiation and favors immature progenitors.^72^ Mechanistically, BALR‐6 interacts with the transcription factor SP1, promoting its activity on leukemogenic targets. Its expression is particularly elevated in *KMT2A*::*AFF1*‐positive cell lines, and it decreases following treatment with the BET inhibitor I‐BET151, suggesting *BALR‐6* is transcriptionally regulated downstream of KMT2A fusions.^72^

In a complementary approach, Kobrossy et al. (2024) introduced a homozygous loss‐of‐function mutation in *KMT2A* in induced pluripotent stem cells, recapitulating epigenetic hallmarks of *KMT2A*‐r leukemia. The resulting increase in H3K4 trimethylation at promoters of leukemic stem cell maintenance genes was accompanied by upregulation of the lncRNA *HOTTIP*, a known activator of *HOXA* genes.^73^ These findings suggest that *KMT2A* rearrangements may exert dominant‐negative effects on wild‐type *KMT2A* function, driving aberrant chromatin states that favor leukemogenesis.

Collectively, these discoveries frame lncRNAs not as bystanders but as key players in the deregulation brought by *KMT2A* rearrangements. Whether modulating chromatin, fine‐tuning transcription, or influencing drug response, lncRNAs such as *LAMP5‐AS1*, *BALR‐2*, and *BALR‐6* reveal themselves as both markers and drivers of oncogenic programs.

### 3.3 miRNAs

miRNAs are small ncRNAs, usually around 22 nucleotides in length, that fine‐tune gene expression post‐transcriptionally.^20^, ^74^ Typically, they bind to the 3′ untranslated region (UTR) of mRNAs, suppressing translation or inducing degradation.^53^, ^75^ Although best known for gene silencing, miRNAs can also enhance translation under specific cellular conditions.^53^, ^74^, ^75^, ^76^ It is predicted that a majority of the human transcriptome is subject to miRNA regulation, underscoring their essential role in fundamental biological processes such as embryonic development, cellular differentiation, and proliferation.^53^ These small ncRNAs have been implicated in the pathogenesis of various cancers, including *KMT2A*‐r ALL, where deregulated miRNA expression contributes to the aberrant gene expression that drives leukemia development.^77^, ^78^, ^79^, ^80^, ^81^, ^82^

Among the first identified miRNAs were miR‐128b and miR‐221, both downregulated in primary KMT2A‐r samples when compared to other subtypes.^83^ Restoring the expression of either miRNA in RS4;11 and SEM cell lines enhanced glucocorticoid‐induced apoptosis, with combined expression showing an additive effect. Notably, their reintroduction also increased vulnerability to serum starvation, suggesting broader roles in stress response and drug sensitivity. Mechanistically, while miR‐128b directly targets *KMT2A::AFF1* and *AFF1::KMT2A*, miR‐221 regulates *CDKN1B*, a cell cycle regulator associated with quiescence and resistance that is upregulated by both *KMT2A::AFF1* and wild‐type *KMT2A*.^83^

In addition to this deregulation, a naturally occurring A13G mutation was also reported in pri‐miR‐128b, found in RS4;11 cells and patient samples, which has been shown to impair its processing into mature miRNA, reducing its effectiveness in restoring glucocorticoid sensitivity.^84^ Additional miRNAs—including miR‐128b's isoform—miR‐128a and miR‐130b are upregulated in *KMT2A*‐r leukemia.^79^ Silencing these miRNAs in cell lines led to a reduction in cell proliferation and increased cell death, partly via the loss of repression on tumor suppressor genes *NR2F6* (miR‐128a) and *SGMS1* (miR‐130b).^79^

Another set of miRNAs identified in *KMT2A* rearrangements is *miR‐143*, *miR‐142*, and *miR‐205*, which have been implicated in the regulation of *KMT2A::AFF1*.^85^ Among these, *miR‐143*, which is downregulated in *KMT2A*‐r ALL, had the most significant impact on *KMT2A::AFF1* protein levels and its target genes, *HOXA9* and *HOXA7*. Induced *miR‐143* expression in *KMT2A::AFF1* cell lines inhibited cell growth and increased apoptosis, negatively affecting leukemia cell proliferation—an effect that was not observed in a *KMT2A::AF9* cell line. Furthermore, *KMT2A::AFF1* was shown to increase the methylation status of the *miR‐143* locus, an effect not observed in *KMT2A::AFF1*‐negative cell lines and blasts.^85^ Moreover, induced expression of *miR‐142‐3p*, the strand derived from the 3′ arm of the precursor hairpin of *miR‐142*, suppressed cell proliferation and increased apoptosis in RS4;11 leukemic cells while also reducing *KMT2A::AFF1* mRNA and expression of *HOXA9*, *HOXA7*, and *HOXA10*.^77^ Conversely, its inhibition led to increased *KMT2A::AFF1* mRNA levels and upregulation of its downstream targets. Strikingly, the fusion protein itself binds to the *miR‐142* promoter, actively repressing its expression.^77^

The regulation of *HOXA* genes is a central theme in *KMT2A*‐rearranged leukemia, with multiple miRNA contributing to their modulation.^77^, ^86^, ^87^, ^88^ One of these is *miR‐27a*, which appears as a regulator of *KMT2A::AFF1* and *AFF1* in RS4;11 cells.^86^ This miRNA disrupts *KMT2A::AFF1* binding to the *HOXA9* promoter, while also downregulating *MEIS1* and impairing cell viability and clonogenicity, placing *miR‐27a* as a functional barrier to *HOXA*‐driven leukemogenesis.^86^ While *miR‐27a* acts to constrain *HOXA* activation, *miR‐196b* appears to play a more complex and context‐dependent role.^86^, ^87^, ^88^, ^89^ One of the most upregulated miRNA in *KMT2A*‐r patients,^89^ *miR‐196b* is induced by both wild‐type and fusion KMT2A proteins.^88^ Encoded within the *HOXA* cluster itself, *miR‐196b* is not unique to *KMT2A*‐driven leukemia but is also observed in *T‐ALL* and other leukemias with aberrant *HOXA* activation.^89^ This miRNA exhibits a context‐dependent function in *KMT2A*‐r ALL, since it can regulate both oncogenes and tumor suppressors. Interestingly, in mouse models, its overexpression initially delayed leukemia onset but later promoted a more aggressive phenotype in secondary transplants, suggesting a dual and stage‐specific role in leukemogenesis.^87^

Delving into another layer of *HOX/MEIS1* regulatory networks, there is the *miR‐17‐92* polycistron, a cluster encoding multiple miRNAs within a single *pri‐*miRNA, which is aberrantly expressed in *KMT2A*‐r ALL and plays a significant role in the regulation of *HOX* pathways.^61^ This *miR‐17‐92* cluster, encoding seven miRNAs, is upregulated in *KMT2A*‐r ALL through both DNA amplification and direct transcriptional activation by *KMT2A* fusions.^61^ Overexpression of this cluster promotes survival, suppresses apoptosis, and blocks differentiation in mouse bone marrow progenitors, inhibiting *B*‐cell development at the pro‐*B* to pre‐*B* transition, especially in cooperation with *KMT2A* fusions.^61^ Within this cluster, *miR‐17‐5p* and *miR‐19a‐3p* both target and negatively regulate *PKNOX1*, a factor that competes with *MEIS1* in forming *PBX‐HOX* complex interactions, favoring the formation of the *MEIS1‐PBX‐HOX* complex, which is essential for leukemic transformation.^90^ Silencing these miRNAs in RS4;11 cells significantly reduces proliferation, an effect that is likely mediated through *PKNOX1*, since its exogenous overexpression promotes differentiation and restrains leukemic growth.^90^

Recent transcriptomic analyses after cytotoxic treatment also revealed a dynamic shift in miRNA expression depending on drug and cell lineage, highlighting several miRNAs in key biological processes, including chemotherapy response and tumor progression.^80^ In RS4;11 cells, cytarabine treatment altered *miR‐370* and *let‐7e* expression, while dexamethasone induced changes in 28 distinct miRNAs. In SEM cells, cytarabine promoted the deregulation of miR‐30a, miR‐370, and miR‐34b, while miR‐31, miR‐410, let‐7b, and let‐7c were the most affected after dexamethasone treatment. These miRNAs intersect with pathways of tumor suppression, chemotherapy resistance, and leukemia progression.^80^ Out of these highlighted miRNAs, *let‐7b* stands out.^78^, ^91^ This miRNA is epigenetically repressed in *KMT2A*‐r leukemia, and its induced expression has been shown to impair leukemic cell growth.^78^ In *KMT2A::AFF1*‐rearranged cells, the downregulation of *let‐7b* contributes to the overexpression of *HMGA2*, a chromatin remodeling factor and transcriptional repressor of *CDKN2A*.^91^ The *KMT2A* fusion protein not only suppresses *let‐7b* but also activates *HMGA2* transcription, a potent transcriptional repressor of the cell cycle inhibitor encoded by *CDKN2A*.^91^

Restoring *let‐7b* expression presents therapeutic potential but requires combined targeting of multiple suppression mechanisms, since this miRNA is suppressed by both hypermethylation and direct inhibition by *KMT2A* fusion proteins.^91^ A dual treatment approach using 5‐azacytidine (a demethylating agent) and netropsin (an *HMGA2* inhibitor) successfully reactivated *CDKN2A* and reduced leukemic cell viability in vitro. This combinatorial strategy opens the possibility of targeting *let‐7b* in interventions for the treatment of *KMT2A*‐r leukemia, even though further assays are required to make this sort of treatment viable.^91^

As a whole, miRNAs seem to have a central role in shaping the molecular landscape of *KMT2A*‐rearranged leukemia. By regulating key fusion transcripts, *HOX* gene expression, and drug response pathways, miRNAs not only provide valuable insight into the mechanisms driving leukemogenesis but also emerge as promising candidates for therapeutic intervention.^61^, ^78^, ^90^, ^91^ As research advances, targeting specific miRNA networks may offer new strategies to overcome resistance and improve outcomes in this high‐risk leukemia subtype.

### 3.4 eRNAs

Our understanding of enhancer genomic regions and their role in gene expression has shifted dramatically with the realization that many of these regions are transcriptionally active and generate non‐coding RNAs, defined as enhancer RNAs (eRNAs).^92^, ^93^, ^94^ This discovery emerged from the observation that most extragenic transcription sites correspond to genomic regions with enhancer‐type chromatin signatures and that these regions may be bidirectionally transcribed by RNA Polymerase II.^92^, ^93^

Recent evidence suggests that many, if not all, functional enhancers are transcribed and functional studies have demonstrated that the knockdown of different eRNAs leads to downregulation of their nearby coding genes, providing evidence for their role in gene activation.^93^, ^95^, ^96^ The transcription of eRNAs has been shown to reinforce cell‐type‐specific and stimulus‐responsive gene expression programs and their dysregulation has been implicated in various diseases, including cancer, where they contribute to aberrant transcriptional networks, making them promising targets for therapeutic intervention.^97^

KMT2A fusion proteins have been demonstrated to induce the expression of the SEELA eRNA cluster through direct binding at the SEELA locus and through epigenetic activation mediated by HOXA9 and HOXA10, downstream targets of the KMT2A oncogene.^98^ This cluster, comprising SEELA1 and SEELA2 variants, interacts directly with histone H4 to enhance chromatin binding and promote enhancer activity. The SEELA cluster regulates the nearby oncogene *SERINC2*, which plays a critical role in disease progression by modulating sphingolipid metabolism. Knockdown of SEELA1 or SEELA2 variants disrupts the transcriptional activation of SERINC2, reducing its expression and impairing leukemic cell proliferation while having minimal impact on neighboring genes such as *FABP3* and *TINAGL1*, confirming specificity in its regulation.^98^

Another eRNA that has recently emerged as a fundamental regulator in *KMT2A*‐rearranged leukemia is *Myrlin*, transcribed from an enhancer near *MYB*.^60^ This eRNA plays a central role in recruiting the KMT2A complex to the *MYB* locus. It interacts directly with KMT2A, serving as a scaffold to facilitate the recruitment of the KMT2A complex and promoting histone H3 lysine 4 trimethylation (H3K4me3), a hallmark of active transcription. Chromatin immunoprecipitation (ChIP) and RNA immunoprecipitation (RIP) assays demonstrated that depletion of this eRNA disrupts KMT2A binding at the *MYB* promoter, reducing MYB expression and impairing leukemic cell proliferation in *KMT2A*‐rearranged leukemia.^60^

These findings collectively highlight the role of eRNAs as modulators of transcriptional regulation in both physiological and pathological contexts.^60^, ^98^ In leukemia, eRNAs not only orchestrate enhancer–promoter interactions but also serve as critical regulators of oncogenic transcriptional networks.^60^, ^98^ As our understanding of eRNAs deepens, their potential as biomarkers and targets for precision medicine continues to grow, offering promising avenues for the development of innovative treatments for *KMT2A*‐rearranged leukemia.

## 4 CONCLUSION

Post‐genomic medicine has opened new opportunities to explore various data dimensions in tumorigenesis, enabling a more comprehensive identification of genetic signatures that can improve the discovery of prognostic biomarkers and therapeutic targets.^9^, ^31^, ^53^

**FIGURE 2:**
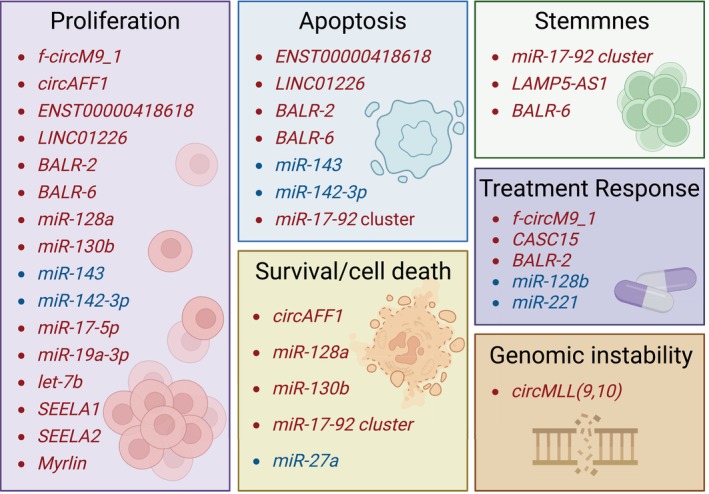
Association of deregulated non‐coding RNAs with key biological processes in *KMT2A*‐rearranged acute lymphoblastic leukemia (*KMT2A*‐r ALL). Downregulated ncRNAs are shown in red, while upregulated ncRNAs are shown in blue.

Here, we critically showed evidence of the ncRNAs in *KMT2A*‐r ALL context as a new layer to be explored under tumoral deregulation perspective (Figure 2). Despite the high diversity of the *KMT2A*‐r involved in ALL, we reported ncRNA sharing among molecular networks that represent promising therapeutic targets or progression biomarkers.^69^, ^77^, ^78^, ^86^ On the other hand, *KMT2A*‐r subtype‐specific expression patterns highlight candidates for diagnostic classification and risk stratification in patients with *KMT2A*‐r ALL.^28^, ^69^

Rather than mere transcriptional byproducts observed in tumoral cells and experimental models, the ncRNAs emerge as new frontiers in *KMT2A*‐r ALL studies. However, our assessment of the current state of knowledge regarding ncRNA reveals important questions to fill gaps about this theme: (i) increased studies encompassing larger numbers of cell lines covering more diverse *KMT2A*‐r TPGs, (ii) decoding the regulatory networks of ncRNAs in more comprehensive *KMT2A*‐r patient cohorts, and (iii) identifying unique genetic signatures of the ncRNA in *KMT2A*‐r tumor progression. In addition, the lack of studies exploring piRNAs in *KMT2A*‐r ALL underscores an important knowledge gap that should be addressed in future investigations.

Beyond these mechanistic aspects, future perspectives may include the exploration of ncRNAs as potential biomarkers or indirect therapeutic targets, either through modulation of their expression or by repurposing drugs known to influence ncRNA‐regulated pathways. Although no compounds currently target ncRNAs specifically in *KMT2A*‐r ALL, advances in this area could pave the way for more precise and personalized therapeutic strategies. Addressing these questions will not only deepen our understanding of *KMT2A*‐r leukemia but also provide the foundation for innovative therapeutic strategies that contribute to more effective and personalized treatments in the future.

## AUTHOR CONTRIBUTIONS

**Maria Augusta Poersch:** Writing – original draft; writing – review and editing; conceptualization. **Ana Carolina Rodrigues:** Writing – review and editing. **Priscila Elias Ferreira Stricker:** Writing – review and editing. **Alexandre Luiz Korte Azevedo:** Writing – review and editing. **Daniel Pacheco Bruschi:** Writing – review and editing. **Jaqueline Carvalho de Oliveira:** Supervision; conceptualization; writing – review and editing.

## FUNDING INFORMATION

This study was supported by the Coordenação de Aperfeiçoamento de Pessoal de Nível Superior (CAPES; scholarship support 001) and the Conselho Nacional de Desenvolvimento Científico e Tecnológico (CNPq; research funding).

## CONFLICT OF INTEREST STATEMENT

All authors declare that they have no competing interests.
